# Intranasal administration of human mesenchymal stromal cell-derived small extracellular vesicles delays disease progression in the SOD1(G93A) mouse model

**DOI:** 10.1186/s13041-026-01288-0

**Published:** 2026-03-03

**Authors:** Ryosuke Hirota, Karen L. Lankford, Masahito Nakazaki, Masayuki Toyoshima, Jeffery D. Kocsis

**Affiliations:** 1 Department of Neurology, Yale University School of Medicine, New Haven, Connecticut 06510 USA; 2 Center for Neuroscience and Regeneration Research, VA Connecticut Healthcare System, (127A), West Haven, Connecticut 06516 USA; 3 Department of Orthopaedic Surgery, Sapporo Medical University, Sapporo, 060-8543 Japan; 4 Department of Neural Regenerative Medicine, Research Institute for Frontier Medicine, Sapporo Medical University School of Medicine, Sapporo, Hokkaido 060-8556 Japan; 5 Division of Regenerative and Advanced Therapy, Nipro Corporation, Osaka, Osaka 531-8510 Japan

**Keywords:** Amyotrophic lateral sclerosis, Mesenchymal stromal cell, Small extracellular vesicles, Intranasal administration, Exosome

## Abstract

Amyotrophic lateral sclerosis (ALS) is a fatal neurodegenerative disease characterized by progressive motor neuron loss, with no established disease-modifying therapy. Mesenchymal stem/stromal cells (MSCs) have been reported to exert neuroprotective effects in models of injury and disease, acting primarily through release of small extracellular vesicles (sEVs). MSC-derived sEVs (MSC-sEVs) have therefore attracted attention as a potential cell-free therapeutic approach for treating neurological conditions such as ALS. Because MSC-sEVs can cross both the nasal epithelial barrier and blood–brain barrier to reach the central nervous system (CNS), intranasal administration represents an attractive approach for repeated delivery of MSC-sEVs for long-term administration. In this study, we administered bone marrow-derived MSC-sEVs or vehicle intranasally to a SOD1(G93A) transgenic mouse model of ALS; the large majority of the sEVs had surface markers for exosomes. Dosing was for three consecutive days per week beginning one day after onset of neurological symptoms and continuing until a moribund state. Neurological score and body weight were recorded daily. Although total survival time and post-onset survival duration were not significantly prolonged by MSC-sEV treatment, MSC-sEV treatment significantly delayed progression from a mild symptom phase (NeuroScore 1) to more severe symptoms (NeuroScore 2) compared with vehicle-treated controls and showed a trend toward slower weight loss. These findings indicate that intranasal administration of MSC-sEVs can delay functional deterioration and prolong the mild impairment stage in an ALS mouse model. If translatable to human patients, such preservation of neurological function could represent a clinically meaningful outcome.

**Supplementary Information:**

The online version contains supplementary material available at 10.1186/s13041-026-01288-0.

Amyotrophic lateral sclerosis (ALS) is a fatal neurodegenerative disorder characterized by the progressive degeneration and loss of motor neurons in the primary motor cortex, brainstem and spinal cord. At present, no therapy capable of fundamentally altering progression has been established [1]. Currently approved treatments provide only limited effects on disease progression and survival. Therefore, there is a strong unmet need for treatments that delay neurological deterioration and preserve patients’ quality of life (QOL). Transgenic SOD1(G93A) mice reproduce many of the key clinical and pathological features of human ALS, including progressive motor impairment, motor neuron loss, disruption of neuromuscular junctions, and body weight loss, and are among the most widely used animal models for ALS research [2]. In SOD1(G93A) mice, neurological function declines rapidly after symptom onset, making it particularly suitable for evaluating whether interventions can delay disease progression.

In recent years, therapies based on mesenchymal stem/stromal cells (MSCs) and their secreted factors have attracted attention as promising treatment approaches for neurodegenerative diseases such as ALS. In particular, small extracellular vesicles (sEVs) derived from MSCs have emerged as a potential cell-free modality capable of exerting neuroprotective effects in ALS models [3, 4]. Moreover, MSC-sEVs delivered by intravenous or intranasal routes have been reported to reach the central nervous system [5], making them an attractive therapeutic modality for neurological disorders. In addition, accumulating evidence suggests that bone marrow–derived MSC-sEVs selectively accumulate in lesion sites in models of stroke and neurodegenerative diseases [6], indicating a potential for targeted delivery of disease-modifying agents.

In the present study, we investigated the therapeutic efficacy of intranasal administration of human bone marrow–derived MSC-sEVs in SOD1(G93A) mice after symptom onset. MSC-sEVs were isolated from cultured human bone marrow MSCs by differential centrifugation and evaluated for purity as described previously [7] and administered intranasally on three consecutive days each week after onset of neurological symptoms (Fig. 1a). Male SOD1(G93A) mice were randomized into treatment groups prior to symptom onset. Treatment responses were evaluated based on longitudinal analysis using a standardized neurological scoring system for this model and body weight changes by investigators who were blinded to the treatment condition until the end of the study (Fig. 1a). More detailed treatment protocols and outcome assessments are provided in the Supplemental Material. Timing of symptom onset and survival duration varied significantly for both the control and MSC-sEVs–treated groups, with no significant difference between groups (Fig. 1b). Although both total survival time (Fig. 1c) and survival time post symptom onset (Fig. 1d) showed a trend toward longer survival time in the MSC-sEV treatment group than in the PBS treatment group, these differences did not reach statistical significance. In contrast, the duration of the mild disease phase, defined as NeuroScore 1, was significantly longer in the MSC-sEVs group compared with the PBS group (*p* = 0.0432) (Fig. 1e), indicating a slowing of progression to more severe impairment. Furthermore, the duration of the mild impairment phase (NeuroScore 1) showed a strong positive correlation with total survival time (Fig. 1f), supporting the trend toward increased survival time in the MSC-sEVs–treated condition.

**Fig. 1:**
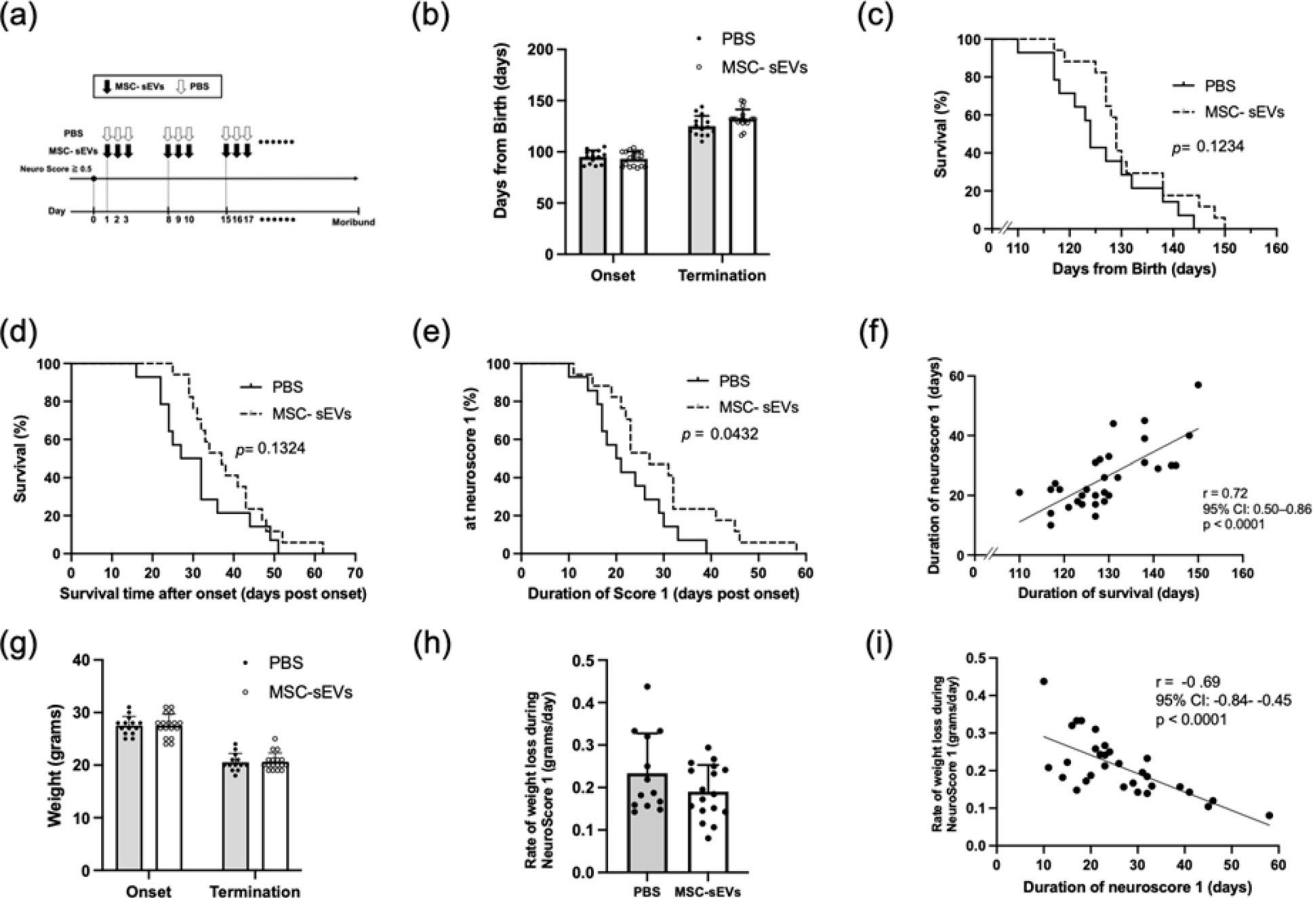
(**a**) SOD1(G93A) mouse treatment protocol timeline. Intranasal PBS or MSC-sEVs treatment was initiated 1 day after the onset of neurological symptoms and delivered on 3 consecutive days per week until the animal was moribund. (**b**) Time of symptom onset and terminal state for PBS (grey bars) and MSC-sEVs (white bars) treatment groups. The mean age at symptom onset was 95.0 ± 6.4 days in the PBS group and 93.2 ± 7.2 days in the MSC-sEVs group. The mean total survival duration was 126.1 ± 9.9 days and 131.8 ± 9.3 days, respectively. No significant differences were found in symptom onset or total survival time between the two randomly assigned treatment groups. (**c**) Kaplan–Meier plots of total survival duration in the PBS (solid line) and MSC-sEVs (dashed line)–treated groups. No significant difference was detected by the log-rank test (*p* = 0.1234). (**d**) Kaplan–Meier plots of survival time after neurological symptom onset in the PBS and MSC-sEVs–treated groups. No significant difference was detected by the log-rank test (*p* = 0.1324). (**e**) Kaplan–Meier plots showing the duration of the mild disease phase (NeuroScore 1) in the PBS and MSC-sEVs–treated groups. A significantly prolonged duration of the mild disease phase was observed in the MSC-sEVs group compared with the PBS group (log-rank test, *p* = 0.0432). (**f**) Dot plot showing the correlation between the duration of the mild disease phase (NeuroScore 1) and total survival time for individual animals. A strong positive correlation was observed (r = 0.72, 95% CI, 0.50–0.86, *p* < 0.0001). (**g**) Body weight at neurological symptom onset and at the terminal stage in the PBS and MSC-sEVs–treated groups. The mean body weight at symptom onset was 27.5 ± 1.7 g in the PBS group and 27.5 ± 2.2 g in the MSC-sEVs group. The mean body weight at the terminal stage was 20.5 ± 1.7 g and 20.6 ± 1.7 g, respectively. (**h**) Average weight loss per day from symptom onset (NeuroScore 1) to disease progression (NeuroScore 2) in the PBS and MSC-sEVs–treated groups. The average daily body weight loss was 0.23 ± 0.09 g in the PBS group and 0.19 ± 0.06 g in the MSC-sEVs group during the NeuroScore 1 phase. (**i**) Dot plot showing the correlation between average weight loss per day from symptom onset (NeuroScore 1) to disease progression (NeuroScore 2) and the duration of the mild disease phase (NeuroScore 1). A significant negative correlation was observed (r =  − 0.69, 95% CI, − 0.84 to − 0.45, *p* < 0.0001)

Body weights for SOD1(G93A) mice decreased significantly during the course of the disease in both control and MSC-sEVs–treated animals, with no significant difference in body weight at symptom onset or termination date between the two groups (Fig. 1g). Rates of weight loss showed a trend toward slower weight loss in MSC-sEV–treated mice compared with controls, although this difference was not statistically significant (Fig. 1h). Notably, the rate of weight loss during the mild symptomatic phase negatively correlated with the duration of NeuroScore 1 (Fig. 1i), suggesting a link between more rapid weight loss and more rapid progression of neurological symptoms.

The results of this study demonstrate that intranasal administration of human bone marrow–derived MSC-sEVs initiated after symptom onset can slow disease progression in SOD1(G93A) mice, prolonging the mild symptomatic phase of the disease, an outcome that could have meaningful clinical implications for QOL if translatable to ALS patients. The observed trends toward longer survival time and slower weight loss in the MSC-sEVs–treated group suggest that a larger sample size or more optimized treatment protocol might produce a statistically significant improvement in longevity. However, further study will be required to identify optimal dosing regimens.

The therapeutic effects of human bone marrow MSC-sEVs observed in this study are consistent with previous reports using MSC-based therapies and MSC-sEVs from other sources. We have previously shown that a single intravenous administration of bone marrow-derived MSCs slowed symptom progression in a SOD1(G93A) transgenic ALS rat model, protected motor neurons, and restored blood–spinal cord barrier (BSCB) integrity [8]. Moreover, repeated administration of MSCs was more effective in delaying motor function decline and also extended survival in this model [9], suggesting that the therapeutic efficacy of MSC treatment may depend on the continued presence of MSCs in the system. Other investigators [10] showed that infusions of adipose MSCs had a similar therapeutic effect on disease progression and survival in a SOD1(G93A) ALS mouse model. Consistent with a large body of data indicating that the therapeutic effects of MSCs are mediated not by engraftment or differentiation of transplanted cells, but rather by sEVs secreted by MSCs [11], repeated intravenous infusions of adipose-derived MSC-sEVs (AD-MSC-sEVs) [4] had similar therapeutic effects in a SOD1(G93A) ALS mouse model as infusion of the parent cells [10]. Furthermore, intranasal delivery of AD-MSC-sEVs every 4 days to SOD1(G93A) mice resulted in delayed progression of neurological symptoms from mild to more severe impairment (Paw Grip Endurance Test), with only a trend toward increased longevity [4]. These results were similar to the effects we observed for intranasal delivery of bone marrow derived MSC-sEVs three consecutive days per week. Taken together, these results suggest that the therapeutic effects of MSCs in rodent models of ALS are mediated by sEV cargoes present in both adipose and bone marrow derived MSC-sEVs, and that intranasal treatment can be an effective route of delivery of therapeutic sEVs in ALS models. Furthermore, the observation that two different treatment schedules resulted in statistically significant delays in disease progression in SOD1(G93A) mice and shows that MSC-sEVs can be therapeutically effective across different dosing schedules, indicating that a broader range of dosing protocols may be effective for ALS-like conditions.

The mechanism of action of MSC-sEVs in ALS models is unclear. However, systemically administered MSC-sEVs localize to affected regions of the brain and spinal cord of SOD1 mice [3], arguing for a direct effect on the CNS, and previous studies have implicated both motor neurons [12] and macrophages/microglia [13] as direct targets of MSC-sEVs in rodent ALS models. In spinal cord injured rats, systemically delivered human MSCs-sEVs are selectively taken up by M2 macrophages and downregulate inflammatory cytokines [14], suggesting that the anti-inflammatory properties of MSC-sEVs [15] may play a role in slowing disease progression. The anti-inflammatory effects may be mediated by bioactive cargoes within MSC-sEVs, including microRNAs, proteins and lipids [16, 17]. MSC-sEVs contain microRNAs that regulate glial activation and inflammatory signaling, as well as proteins with anti-inflammatory and neurotrophic properties. In addition, lipid components of sEV membranes have been implicated in modulating immune responses and cell–cell communication in the CNS [16]. Although the present study did not identify individual molecular contributors, these cargo-mediated mechanisms may collectively underlie the therapeutic effects observed in this ALS model.

The present findings support the therapeutic potential of intranasally delivered MSC-sEVs for treatment of ALS and lay a foundation for further translational and mechanistic studies. Intranasal administration offers substantial practical advantages for treatment of chronic or progressive diseases, as it imposes minimal physical burden on patients and does not require specialized medical equipment or training, making repeated and long-term administration feasible in a home setting.

## Supplementary Information

Below is the link to the electronic supplementary material.

Supplementary Material 1

## Data Availability

The data that support the findings of this study are available from the corresponding author upon reasonable request.
