# Supplementary material for: Intranasal administration of human mesenchymal stromal cell-derived small extracellular vesicles delays disease progression in the SOD1(G93A) mouse model
Source: Mol Brain. 2026 Mar 3;19:22. doi: 10.1186/s13041-026-01288-0 (PMC13063686; doi:10.1186/s13041-026-01288-0)
Supplement: Supplementary file 1 — Supplementary Material 1 [file 13041_2026_1288_MOESM1_ESM.docx]

**Online Supplementary Material for**

**Intranasal Administration of Human Mesenchymal Stromal Cell-derived Small Extracellular Vesicles Delays Disease Progression in the SOD1(G93A) Mouse Model Authors:**

Ryosuke Hirota ^1, 2, 3, 4^, Karen L Lankford ^1, 2^, Masahito Nakazaki ^1, 2, 4^, Masayuki Toyoshima^1, 2, 5^ Jeffery D. Kocsis ^1, 2^

**Affiliations:**

1, Department of Neurology, Yale University School of Medicine, New Haven, Connecticut, 06510, USA;

2, Center for Neuroscience and Regeneration Research, VA Connecticut Healthcare System, West Haven, Connecticut, 06516, USA;

3, Department of Orthopaedic Surgery, Sapporo Medical University, Sapporo 060-8543, Japan

4, Department of Neural Regenerative Medicine, Research Institute for Frontier Medicine, Sapporo Medical University School of Medicine, Sapporo, Hokkaido, 060-8556, Japan;

5, Division of Regenerative and Advanced Therapy, Nipro Corporation, Osaka, Osaka, 531-8510, Japan

**ALS model**

All animal experiments were carried out in accordance with National Institutes of Health guidelines for the care and use of laboratory animals and the VA Connecticut Healthcare System Institutional Animal Care and Use Committee (IACUC) approved all animal protocols.

Male transgenic mice overexpressing the human superoxide dismutase 1 (SOD1) gene carrying the Gly93–Ala mutation (SOD1(G93A)) (strain designation B6SJL–TgN[SOD1–G93A]1Gur, stock number 002726) were obtained from The Jackson Laboratory (Bar Harbor, ME, USA) and housed under controlled environmental conditions, including regulated temperature and humidity, a 12 h light/dark cycle, and ad libitum access to food and water, with continuous veterinary supervision.

**Preparation of MSC-sEVs**

Human bone marrow–derived mesenchymal stem cells (MSCs) were purchased from a commercial supplier (Lonza, Allendale, NJ, USA) and maintained in Dulbecco’s Modified Eagle Medium (DMEM) supplemented with 10% fetal bovine serum (FBS), L-glutamine, and penicillin/streptomycin. Cells were expanded and passaged up to six times once they reached approximately 70–80% confluence. Following the final passage, cultures were washed with phosphate-buffered saline (PBS) and transferred to serum-free medium for the collection of small extracellular vesicles (sEVs) and isolation via differential centrifugation and characterization as described previously (1). Protein content was measured by Bradford assay for all samples, enrichment of exosome-specific markers Alix, CD63, and CD9 were confirmed by Western blot, and size distribution of particles in hMSC-sEVs fractions was evaluated by nanoparticle tracking analysis (NTA) for some samples as described previously (1).

**Experimental protocol**

60 Male mice were randomly assigned to MSC-sEVs or PBS control treatments and underwent desensitization to handling and daily assessments of motor function and body weight beginning at postnatal day 56. Four of the initial 60 animals were euthanized prior to start of treatment or shortly after symptom onset due to fighting injuries.

One day after the onset of lower limb motor symptoms on either one or both sides (NeuroScore 1, see below), 12 µL of phosphate-buffered saline (PBS) or 3 µg of MSC-sEV protein suspended in 12 µL of PBS was delivered intranasally to lightly restrained awake mice. Delivery was alternated between the left and right nostrils (2–3 µL per nostril). Treatments were repeated on three consecutive days each week and continued until animals reached the moribund stage (NeuroScore 4) and were euthanized. Experimenters administering treatments and assessing NeuroScores were blinded with respect to the treatment condition until data collection was completed.

**Symptom Assessment**

Neurological function was evaluated using the NeuroScore system as described by Theo et al. (2), with all assessments performed strictly according to the original criteria. Briefly, scores ranged from 0 (no neurological symptoms) to 4 (terminal stage with loss of forward movement and righting reflex). Intermediate scores reflected progressive hindlimb dysfunction: score 1 indicated early abnormalities (e.g., abnormal hindlimb splay, tremor, or mildly slowed gait); score 2 indicated hindlimb collapse or toe curling with preserved righting within 10 s; and score 3 indicated severe hindlimb dysfunction with preserved righting reflex. Each hindlimb was scored independently, and the mean value was used as the individual NeuroScore. Animals were euthanized when either hindlimb reached a score of 4.

**Weight Loss Assessment**

Animals were weighed to the nearest gram every day after arrival (postnatal day 56). Weight at onset of symptoms (NeuroScore 1), progression to NeruoScore 2 and the termination point (NeuroScore 4) were recorded. Total weight loss during the mild symptomatic phase was determined by subtracting weight at onset of Neuroscore 2 from weight at onset of NeuroScore 1. Rate of weight loss during this phase was assessed by dividing the total weight loss during the mild symptomatic phase (NeuroScore 1) by the duration of this phase.

**Statistics**

All statistical analyses were performed using GraphPad Prism software (version 10.6.1). Survival time and the time of NeuroScore 1 were analyzed using the Kaplan–Meier method, and comparisons between the MSC-sEVs and PBS groups were performed using the log-rank test. The correlation between survival duration (days) and the number of days spent at NeuroScore 1 was assessed using Pearson’s correlation coefficient. For comparisons of body weight between two groups, data were assessed for normality, and either Student’s t-test or the Mann–Whitney U test was applied as appropriate. A p value of < 0.05 was considered statistically significant for all analyses.

**References**

(1) Nakazaki M, Lankford KL, Yamamoto H, Mae Y, Kocsis JD. Human mesenchymal stem-derived extracellular vesicles improve body growth and motor function following severe spinal cord injury in rat. Clin Transl Med. 2023 Jun;13(6):e1284. doi: 10.1002/ctm2.1284. PMID: 37323108; PMCID: PMC10272923.

(2) Hatzipetros T, Kidd JD, Moreno AJ, Thompson K, Gill A, Vieira FG. A Quick Phenotypic Neurological Scoring System for Evaluating Disease Progression in the SOD1-G93A Mouse Model of ALS. J Vis Exp. 2015 Oct 6;(104):53257. doi: 10.3791/53257. PMID: 26485052; PMCID: PMC4692639.
